# Surgery of benign neurogenic tumors in adults: single institution experience

**DOI:** 10.11604/pamj.2014.19.288.4929

**Published:** 2014-11-17

**Authors:** Moulay Brahim Ratbi, Fayçal El Oueriachi, Adil Arsalane, Mohamed Massine El Hammoumi, El Hassane Kabiri

**Affiliations:** 1 Department of Visceral Surgery, Mohamed V Military Academic Hospital, Rabat, Morocco; 2 Department of Thoracic Surgery, Mohamed V Military Academic Hospital, Rabat, Morocco

**Keywords:** Benign neurogenic tumor, mediastinum, adult, surgery

## Abstract

The objective of this work is to review retrospectively our experience with 17 patients presenting with benign neurogenic tumors, managed in the department of thoracic surgery, Mohamed V Military Academic Hospital, Rabat, Morocco. Between 2003 and 2011, seventeen patients were surgically treated for benign neurogenic tumors of the mediastinum, among 112 mediastinal tumors operated during the same period. The mean age of the 17 patients was 46 years, including 11 females and 6 males. The information about clinical presentation, diagnostic procedures, surgical techniques and postoperative follow-up were extracted and analyzed from medical records. Symptoms related to the tumor were found in 13 patients (76,4%). The posterior mediastinum was the principal location (16 cases: 94%). Intraspinal extension was shown through MRI in one case. Surgical extirpation was complete in all patients. There were no tumor-related deaths and no significant complications. There were 13 schwannomas, 2 neurofibromas and 2 ganglioneuromas. Neurogenic tumors of the mediastinum in adults are mostly benign. Their only treatment is surgical extirpation. Video-assisted thoracoscopic resection is currently the best approach in selected patients.

## Introduction

Benign neurogenic tumors are relatively the third most frequent of all mediastinal tumors behind thymomas and probably lymphomas (20% of all primitive tumors of the mediastinum and 75% of the tumors of posterior mediastinum) [1]. They are neoplasms arising from all the components of intrathoracic nervous formations. The rate of malignancy is very low in adults in comparison to pediatric population (6% and 40% respectively) [2]. The posterior mediastinum rich in neurogenic elements (sympathetic chain, intercostal nerves, vagus nerves...) remains their elective localization [3], especially along the costovertebral gutter. The posterolateral thoracotomy has been the traditional surgical approach, in the aim to carry out the complete resection of these tumors; generally benign. Outside certain contraindications, the video-assisted thoracoscopic surgery (VATS) is actually a good alternative for excising such small tumors [4]. The objective of this study was to report our experience in the management of this disease, since its discovery among our patients, until ensuring regular follow-up after surgery, in order to better relate our results.

## Methods

The register of hospitalization of the department of thoracic surgery, at Mohammed V Military Academic Hospital, was consulted to identify all the patients who underwent resection of neurogenic tumors of the mediastinum from January 2003 to December 2010. During this period, 112 patients were operated for mediastinal tumors; seventeen had the histological diagnosis of benign neurogenic tumors. We performed a retrospective study to review their clinical, operative and follow-up characteristics including histological variety. Criteria for eligibility were: patients any sex, aged more than sixteen years old at admission, with only mediastinal localization confirmed by CT scan and no malignant component in the definitive postoperative specimen.

The age of the patients at presentation ranged from 29 to 67 years (mean = 46,2 years). There were 11 female and 6 male patients, constituting a male/female ratio of 0,54. Four patients were asymptomatic; their tumors were chance findings on routine chest X-rays. The clinical past history of all the patients was documented to identify associated diseases possibly related to neurogenic tumor (café au lait spots, hypertension); only one patient had hypertension. The diagnosis procedure was based, in addition to chest roentgenogram, on computed tomography. The MRI was done in four patients who showed a potentially extension into the spinal canal on CT imaging. Serum levels of HVA and VMA in the patient who had hypertension were normal. Epidural analgesia was used for a good postoperative pain control in most our patients. All patients were operated using posterolateral thoracotomy through the fifth intercostal space in 12 patients, and through the fourth one in 5 patients who had an upper mediastinal mass. Neurosurgeon intervention was necessary in one case to achieve a widening of the intervertebral foramen and release the intraspinal component of the tumor. For all patients, surgical procedure was diagnostic and therapeutic. The mean duration of hospital stay was 5,6 days, ranged from 3 to 15 days. The mean follow-up period was 40,6 months (range = 6 to 88 months); it was based on clinical and especially radiological assessment (CT scan).

## Results

**Figure 1:**
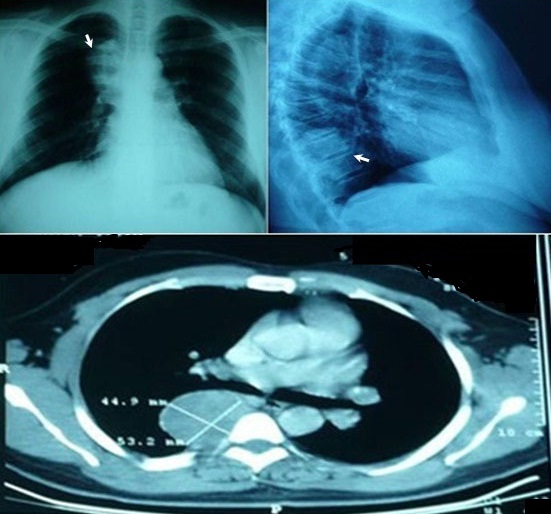
Chest x-ray (top) and computed tomography (bottom) revealing a posterior and well-circumscribed paravertebral mass, corresponding to a schwannoma

**Figure 2:**
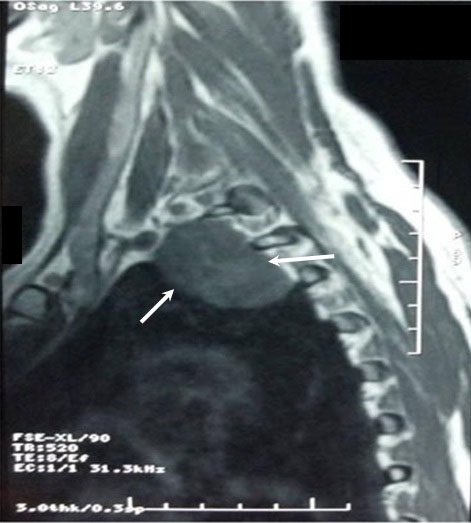
T2-weited imaging of a schwannoma showing typically high signal intensity in the tumor

**Figure 3:**
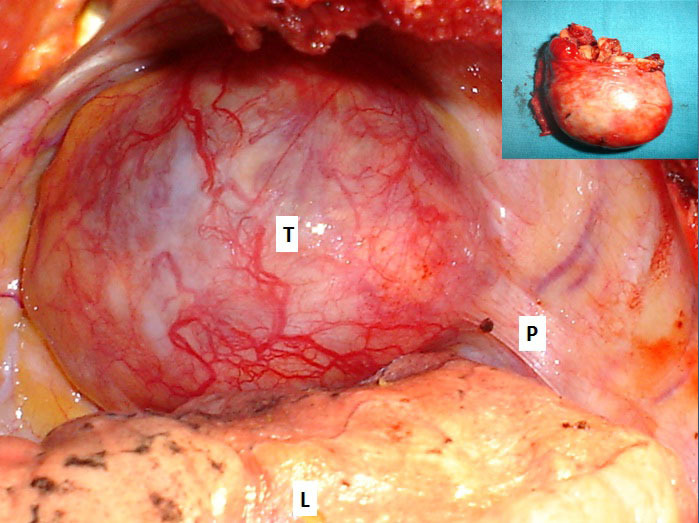
Operative view showing an upper mediastinal mass; (T) tumor; (P) pleura; (L) lung. Miniature: operative specimen

The discovery of the tumor was related to the presence of symptoms in 13 patients: posterior chest pain in 10, intercostal pain in two and respiratory symptoms in four (cough in 3 cases and dyspnea in 1 case). The tumor location was the posterior mediastinum in sixteen cases and the middle mediastinum in one case. It was right-sided in 8 patients and left-sided in 9. The tumor was solid, measuring between 3 and 6,5 cm, the most often well-circumscribed, multilobed in one case. Calcifications and mediastinal lymphadenopathies were found each one in one patient. CT scan was sufficient to suspect diagnosis in all the cases, and to study morphological characteristics of the tumor and its relations with adjacent structures (Figure 1). MRI imaging (Figure 2) done in four cases, it showed a minimal intraspinal extension through the T_3_ - T_4_ intervertebral foramen in one patient. Complete surgical extirpation of the tumor was performed in all patients (Figure 3), including the case that showed an intraforaminal portion. The origin of the tumors was the intercostal nerve in 9 cases, the vagus nerve in 2 cases, the sympathetic chain in 4 cases and it was difficult to determine it in 2 cases.

There were no operative deaths and no major surgical complications. The postoperative morbidity was minor; it consisted in two pneumonias, one phrenic nerve paralysis, two wound infections, six prolonged intercostal neuralgias and paresthesias managed perioperatively with epidural analgesia and further with level 2 analgesics. During all the follow-up period there was no recurrence of the tumor. The benign behavior of the tumor was proved by histopathological exam in all the cases. The tumor size ranged from 2,5x3 cm to 5x6,5 cm. The distribution by histopathological type included 13 schwannomas (76,5%), 2 neurofibromas (11,7%) and 2 ganglioneuromas (11,7%). The tumor with a foraminal extension corresponds to a schwannoma (Table 1).

**Table 1:** Patients and diseases caracteristics

Number		17
Gender		11F /6M
Age (years)		29-67 (mean : 46,2)
Presence of symptoms		13/17
Tumor location	Posterior mediastinum	16
	Middle mediastinum	1
Intraspinal extension		1
Size		Range : 3-6,5
Surgical procedure	Complete extirpation	17/17
	thoracotomy	All patients
	VATS	0
Origin		Intercostal nerve : 9
		Vagus nerve : 2
		Sympathetic chain : 4
		Undetermined : 2
Postoperative complications		Pneumonias : 2
		Phrenic paralesis : 1
		Wound infection : 2
Histology		Schwannoma : 13
		Neurofibroma : 2
		Ganglioneuroma : 2
Recurrence		0/17

## Discussion

In adults, neurogenic tumors of the mediastinum are benign in approximately 92% of cases [5, 6]. They are the most common type of posterior mediastinal tumors. In our series, 16 among the 17 tumors (94%) had a posterior localization; one tumor was located in the middle mediastinum. No clear predominance of sex has been reported in the literature [7]; in our study females were more affected than males (sex ratio = 0,54). Symptoms related to neurogenic tumors are varied; the most frequent are thoracic pain, dorsal or intercostal neuralgia, respiratory symptoms such as cough or dyspnea and Horner's syndrome. Their presence is related to the tumors size and location (the Horner's syndrome is most found in supero-posterior tumors). Neurological symptoms are found when compression or involvement of spinal cord by a tumor exists. In our patients the most frequent symptoms were: chest pain and respiratory symptoms. The asymptomatic form represented approximately ¼ of cases. It has been reported that 10% of these tumors may have an intraspinal extension (Dumbell tumor) [8], and cause signs of medullary compression. Our patient had an intraspinal extension, she was neurologically asymptomatic. Signs related to Von Recklinghaussens disease should be investigated because of their association to multiple neurogenic tumors [9].

In general, the preoperative diagnosis of benign neurogenic tumor of the mediastinum is most often established by radiographic imaging alone. Chest X-ray can diagnose the thoracic topography of the tumor (often posterior) [10]. CT scan provides information about the topography, the size, the density and the enhancement of the tumor following contrast injection, the presence of calcifications and the relations with adjacent structures; what are criteria for the diagnosis and to predict the difficulties of resection [10, 11]. In all our patients the preoperative diagnosis was successfully established by CT scan. MRI should be performed when the tumor is suspected to extend into the spinal canal, so as to determine the longitudinal extent of the tumor in the spine for better neurosurgical procedure [12]. In adults, nerve sheath tumors, such as schwannomas and neurofibromas, are the most frequent followed by the tumors of the autonomic system, such as ganglioneuromas. Tumors arising from the paraganglionic system (paragangliomas) are very rare [7]. Schwannomas, also called neurilemmomas, are the most frequent tumors of nerve sheath, usually developed from the sensory root of an intercostal nerve, and rarely from the phrenic or vagus nerve [13]. Nerve cell tumors of autonomic system are more common in children. Their reported risk of malignancy is between 27% and 76% [14, 15]. The malignancy rate in adults is reported to be between 4% and 12% [7, 16], represented mainly by malignant shwannoma. In our practice, most tumors were derived from the nerve sheaths (88%), among witch shwannoma was the most frequent (76,5%) of all the histological types. Malignant transformation of a pre-existing shwannoma is very rare, explaining that malignancy was found in none of our patients.

The differential diagnoses of neurogenic tumors of the posterior mediastinum are numerous even after CT scan, especially in their cystic form. Bronchogenic cyst, hydatid cyst and lymphangioma can be excluded only in the moment of surgery. Cervico-mediastinal goiters and meningoceles are easily excluded by CT scan [17]. The gold standard in the treatment of benign neurogenic tumors is surgery to achieve complete resection of the tumor. The standard posterolateral thoracotomy is the most used approach. However, in order to preserve the integrity of the respiratory muscles, to minimize postoperative pain, and depending on the location of the tumor; posterior or axillary thoracotomy can be achieved [18]. In our series, surgical excision of the tumor was complete in all our patients. Watchful observation of mediastinal masses is rarely justified because none of the preoperative investigations can completely exclude the malignancy of such lesions. Patients with intraspinal extension of the tumors required a combined thoracic and neurosurgical approach. The objective is to raise or prevent defects related to cord compression. Several neurosurgical techniques were described [19–21] to be performed first, then followed by thoracic procedure. The injury of Adam Kiewickz artery during dissection of the posterior and internal part of the tumor, especially tumors located above Th 7, must be prevented through the identification of its origin and its course by spinal angiography. Benign mediastinal neurogenic tumors are good indications for video-assisted thoracoscopic resection (VATS) [22, 23]. This approach provides an excellent view to easily identify the tumor usually covered by pleura. The posterior mediastinum is the ideal site to VATS approach. The tumor is usually well encapsulated and can be easily mobilized from surrounding structures. The use of endoclips is preferred for neurovascular pedicles of the tumor.

Being minimally an invasive technique, VATS minimizes postoperative pain and complications, reduces the hospital stay and therefore the hospital cost, in addition to its esthetic aspect. Conversion to thoracotomy was rarely reported [23], because of technical difficulties, operative complications or suspicions of malignancy. Contraindications to thoracoscopic approach are relative, including: tumors size more than 6 cm, intraspinal extension, tumors located in the apex of the chest or in the costodiaphragmatic sulcus and malignant tumors. Results of the surgery of benign neurogenic tumors of the mediastinum in adults are related to the quality of resection and the histological type. Indeed, the rate of recurrence after complete resection is almost null. Schwannomas and ganglioneuromas showed good results after surgery, however the risk of malignant transformation should be considered in neurofibromas, especially when associated to Von Recklinghaussens disease [24].

## Conclusion

In conclusion, surgery is the treatment of choice for neurogenic (mostly benign) tumors of the mediastinum in adults. VATS resection is increasingly reported, with convincing results, to be a good alternative in managing benign neurogenic tumors of the thorax in selected patients. Recurrence or the need to an additional therapy should no longer be considered after complete resection. The combined neurosurgical and thoracic approach is the rule in the surgical management of Dumbell tumors.
